# HIV PrEP programmes as a framework for diagnosing and treating HBV infection in adolescents and young adults in KwaZulu-Natal, South Africa

**DOI:** 10.1016/j.jve.2025.100600

**Published:** 2025-06-06

**Authors:** Gloria Sukali, Jacob Busang, Jaco Dreyer, Thandeka Khoza, Marion Delphin, Nonhlanhla Okesola, Carina Herbst, Elizabeth Waddilove, Janine Upton, Janet Seeley, Collins Iwuji, Motswedi Anderson, Philippa C. Matthews, Maryam Shahmanesh

**Affiliations:** a Africa Health Research Institute, KwaZulu-Natal, South Africa; b Division of Infection and Immunity, University College London, Gower Street, London, WC1E 6BT, UK; c The Francis Crick Institute, 1 Midland Road, London, NW1 1AT, UK; d Department of Global Health and Development, London School of Hygiene & Tropical Medicine, London, WC1H 9SH, UK; e School of Nursing and Public Health, University of KwaZulu-Natal, KwaZulu-Natal, South Africa; f Department of Global Health & Infection, Brighton and Sussex Medical School, University of Sussex, Falmer, Brighton, BN1 9PX, UK; g Botswana Harvard Health Partnership, Gaborone, Botswana; h Department of Infectious Diseases, University College London Hospital, Euston Road, London, NW1 2BU, UK

**Keywords:** HIV, HBV, Hepatitis B, South Africa, Infection, Prevention, Prevalence, Diagnosis, Decentralisation, Treatment, PrEP, sexual health, reproductive health, triple elimination

## Abstract

**Background:**

Guidelines for Hepatitis B treatment released by the World Health Organization in 2024 include the potential for use of dual therapy, combining tenofovir with either emtricitabine or lamivudine. These fixed-dose combinations are also used for Pre-Exposure Prophylaxis (PrEP) in people at risk of Human Immunodeficiency Virus (HIV). We hypothesize that pre-existing HIV PrEP programmes can support access to HBV testing and treatment.

**Methods:**

At the Africa Health Research Institute (AHRI) in KwaZulu Natal, South Africa, we evaluated PrEP uptake and retention amongst adolescents and young adults aged 15–30 years. We reviewed HBV status, acceptance of PrEP and retention in follow-up between June 2022–Sept 2024.

**Results:**

15847 adolescents and young adults received an assessment in the community, of whom 3481/15847 (21.9 %) were eligible for sexual health prevention interventions. 3431/3481 (98.6 %) accepted HBV screening, of whom 21/3431 (0.6 %) tested positive for HBsAg. These 21 individuals had not previously been aware of their HBV status, but one was already on antiretroviral therapy for HIV infection. Amongst the others, 16/20 (80 %) were considered eligible for PrEP, and 15/16 started PrEP. When investigating retention in care, among 15 individuals due for a refill, 8/15 (53.3 %) returned at least once.

**Conclusion:**

Sexual reproductive health and PrEP programmes provide an opportunity for HBV testing and treatment. However, attrition from the care cascade at each step highlights the pressing need for interventions that address barriers to sustainable delivery of long-term care.

## 1 Introduction

High profile global goals have been established for the elimination of hepatitis B virus (HBV) as a public health threat, with specific targets for the year 2030.^1^ Nucleos/tide analogue (NAs) agents are offered to those deemed at the highest risk of chronic liver disease to reduce morbidity and mortality, and to reduce transmission. Tenofovir disoproxil fumarate (TDF), the first line agent, is included in the World Health Organisation (WHO) essential medications list, and should be widely accessible and affordable (global benchmark price US $2.40/month).^2^ However, the practical reality is that TDF is not available - or incurs unacceptable out-of-pocket costs - for many high-prevalence populations, including those in the WHO African region, which now account for >60 % of new HBV infections.^2^

In March 2024, the WHO published new guidelines for HBV,^3^ which simplify and widen treatment eligibility criteria, and for the first time include a conditional recommendation for dual therapy when tenofovir monotherapy is not available (either as TDF or as Tenofovir Alafenamide, TAF). This provides flexibility for tenofovir to be prescribed as part of fixed-dose combination therapy, together with either lamivudine (3TC) or emtricitabine (FTC), collectively termed ‘XTC’.^3^ In many settings, Tenofovir/XTC is more affordable and accessible than TDF monotherapy as a result of its widespread procurement for HIV treatment. These fixed-dose combinations are also used as Pre-Exposure Prophylaxis (PrEP) in adolescents/adults who are at risk of HIV acquisition. PrEP therefore offers the combined benefit of HBV treatment and HIV prevention.^4^ Evidence in small cohorts also suggests PrEP could be used as prevention for HBV, when vaccination is not available.^5^ Such approaches may be of particular strength in African settings, where HBV programmes can build on expertise, infrastructure and resources that have been developed for tackling HIV.^6^

In South Africa (SA), HIV and HBV infection are co-endemic.^7^^,^^8^ HIV has been tackled through scale-up of education, screening and treatment, leading to proactive community engagement and robust access pathways to diagnosis and treatment. Antiretroviral therapy (ART) has been available free of charge through the South African government. In comparison, HBV infection has been neglected,^9^^,^^10^ with poor awareness, high stigma, and low access to interventions for prevention, diagnosis and treatment.^11^, ^12^, ^13^

We here describe the results of a collaboration between translational research studies for provision of HIV-PrEP and HBV care pathways, based at the Africa Health Research Institute (AHRI) in KwaZulu-Natal (KZN), South Africa. We aimed to gather preliminary data to explore the prevalence of HBV infection among adolescents and young adults (AYA) engaging with sexual health screening and PrEP services, and to determine the uptake of PrEP for people testing HBV surface antigen (HBsAg)-positive.

## 2 Methods

### 2.1 Study setting and research cohorts

**Fig. 1:**
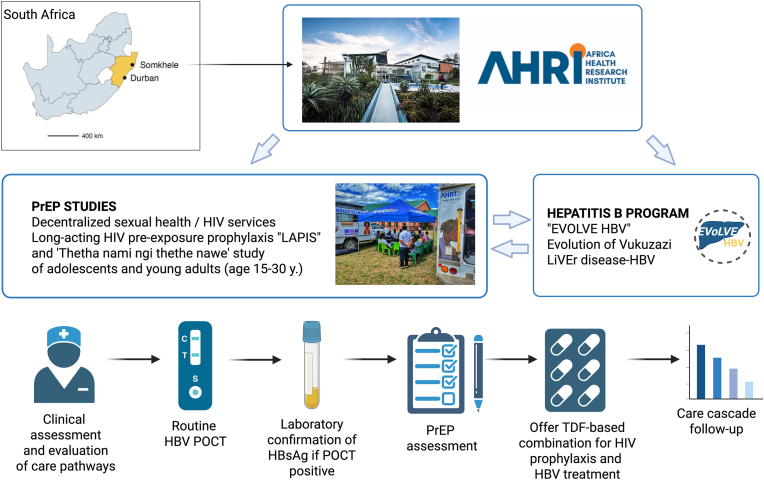
**Schematic structure of studies at the Africa Health Research Institute (AHRI) to investigate frameworks for delivery of sexual health services and HIV pre-exposure prophylaxis (PrEP) as a foundation for delivering testing and treatment for HBV.** A: Location of study sites; map shows South Africa with the KwaZulu-Natal province coloured gold and the two AHRI sites of Durban and Somkhele highlighted. B: Collaboration between PrEP studies and HBV program. C: Pathway offered to adolescents and young adults showing HBV screening, assessment, offer of PrEP and follow-up. Created in BioRender. Matthews, P. (2025); https://BioRender.com/a8bec78.

We developed a collaboration between programmes at AHRI (Fig. 1). The ‘EVOLVE-HBV’ study started in 2023 as a collaboration between AHRI and University College London (UCL) and the Francis Crick Institute in the UK, to describe HBV epidemiology in the KZN population, characterize the clinical and laboratory features of HBV infection, and engage with local communities and healthcare providers to implement sustainable care pathways to diagnosis and treatment.^11^^,^^14^ This study is approved by the University of KwaZulu-Natal (UKZN) Biomedical Research Ethics Committee (BREC) (ref. 00004495/2022) in SA, and UCL ethics committee in the UK (ref. 23221/001 EVOLVE-HBV).

To explore the role and impact of programmes providing HIV PrEP for tackling HBV infection in AYA aged 15–30 years, we used a framework established by ‘*Thetha nami ngithethe nawe*’ (‘Talk to me and I'll talk to you’ in IsiZulu),^15^ which is a stepped-wedge cluster-randomised controlled trial to investigate the effectiveness, implementation and cost effectiveness of peer-led social mobilisation into decentralised HIV and sexual reproductive health (SRH) services, approved by UKZN BREC (ref. 473/2019).^15^ Young people in the community are engaged by peer navigators and offered a needs assessment. Those at risk of sexually transmitted infection (STI) or HIV are referred for review in nurse-led mobile clinics that provide testing and treatment, offer contraception, preconception care, and tailored HIV prevention. Oral daily PrEP is offered to those who are at risk of acquiring HIV, based on national criteria for assessment (Box 1), and willingness to engage with taking daily medication and attending follow-up. *Thetha nami* has also provided a platform for the Long-Acting HIV PrEP (*LAPIS*) study, approved by UKZN BREC (ref. 3735/2021**).** LAPIS is embedded within the second period of the *Thetha nami* trial. It is a cluster-randomised controlled trial of effectiveness and implementation, offering a choice of injectable PrEP, continuous oral daily PrEP, dapivirine vaginal rings, or packs of oral post exposure antiviral prophylaxis for HIV prevention, compared with the offer of oral PrEP alone.

Box 1 **PrEP eligibility and risk assessment questions based on South African National PrEP/ART guidelines [26]**

**Eligible for PrEP**.

1. HIV negative.
2. Willing and able to take a pill every day.
3. Willing to return for 3 monthly follow-up.
4. Understand that PrEP does not protect against pregnancy and STIs (contraception or condoms needed).

**Risk assessment**.

1. Participant at significant risk of acquiring HIV (adolescent and young person who wants PrEP due to self-perceived risk; key population – esp. if adolescent or young: HIV negative MSM or transgender person; engage in transactional sex or sex work; person who inject drugs)?
2. Are they sexually active?
3. Number of partners?
4. Condomless sex in the past 3 months?
5. UPSI with HIV positive or serostatus unknown person past 12 months?
6. Sex under the influence of drugs or alcohol?

Alt-text: Box 1

ART: Antiretrovirals; HIV: Human Immunodeficiency Virus; MSM - men who have sex with men; PrEP - pre-exposure prophylaxis, STI - sexually transmitted infection; UPSI - unprotected sexual intercourse.

### 2.2 Clinical screening, and data collection

We reviewed prospectively collected data of participants in the mobile SRH clinics between June 2022–September 2024. Mobile study clinics follow a monthly schedule visiting different sites in the study area. Clinic attendees are offered HIV counselling and point of care testing (POCT), with immediate information and initiation of ART for those testing HIV positive. Individuals who are HIV negative undergo assessment for PrEP eligibility according to SA National PrEP/ART guidelines (box 1). The current preferred PrEP regimen in SA is a fixed-dose combination of oral TDF/FTC.

All clinic attendees are also offered testing for STIs, with POCT for syphilis, and self-taken vaginal swabs or urine tests for gonorrhoea and chlamydia (**Supplementary methods**). If any of the STIs are positive these individuals receive treatment^16^ and the programme supports partner notification.

Hepatitis B screening is undertaken using POCT in *Thetha nami,* and by taking venous blood for laboratory testing in *LAPIS*. Confirmation tests are conducted using Abbott Architect System (Abbott Park, Illinois, USA).

### 2.3 Sexual health screening and follow-up

**Fig. 2:**
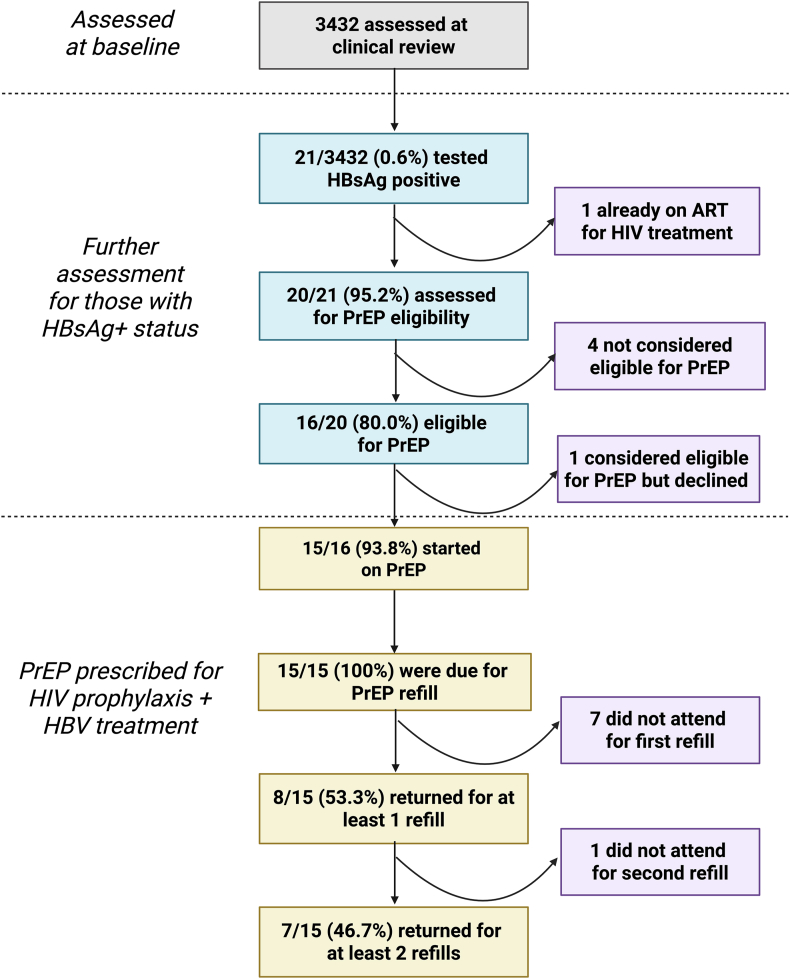
**Care cascade summary of screening, linkage to care, receipt of PrEP and continuation of PrEP for young adults and adolescents accessing sexual and reproductive health services in a rural population in KwaZulu Natal, South Africa.** Flow chart shows the number of people represented in each category, with numbers in purple boxes explaining those not continuing in follow-up. HBsAg: Hepatitis B surface antigen; PrEP: Pre Exposure Prophylaxis (in this case used for HIV prophylaxis and HBV treatment). Created in BioRender. Matthews, P. (2025); https://BioRender.com/gpfqku5.

For individuals who screened positive for HBsAg, we reviewed their progress using an approach based on the care cascade for HBV (Fig. 2), focusing on testing (offer and uptake), referral to study physician, PrEP uptake and return for medication refills. Once participants have been initiated on PrEP, they are scheduled for a mobile clinic appointment one month after PrEP initiation. As per national guidelines, refills and monitoring are every three months thereafter, through mobile clinic appointments^15^; community refills are provided, aiming for continuous PrEP supplies. For those who do not return for a PrEP refill, the clinical and study teams aim to make contact by phone, peers or trackers (providing home visits).

### 2.4 PrEP initiation and follow-up time definitions

● Time 0 - Initiation visit (30 days of PrEP is supplied);
● Time 1 - First PrEP refill; visit 1 month after treatment initiation (90 days PrEP supplied);
● Time 2 - Second PrEP refill; visit 4 months after treatment initiation (90 days PrEP supplied).

## 3 Results

### 3.1 Community acceptance of screening for HBV

Over the time period reviewed (June 2022–Sept 2024), 15,847 AYA received a ‘needs assessment’ by peer navigators in the community, of whom 3481 (21.9 %) were eligible for HIV prevention interventions and referred to the mobile SRH services for clinical review. In this population, 3431 (98.6 %) accepted HBV screening, of whom 21 (0.6 %) tested positive for HBsAg (Table 1; Fig. 2). The majority were female (2019/3431, 59 %), and living in rural areas (2712/3431, 79 %), and the most commonly represented age group was 20–24 years (1295/3431, 38 %).

**Table 1:** Characteristics of adolescents and young adults attending the *‘Thetha nami’* or *‘LAPIS’* study mobile clinics in KwaZulu-Natal for sexual and reproductive health, classified by Hepatitis B surface antigen (HBsAg) status.

Participant characteristic	Total cohort^a^ (N = 3431)	HBsAg positive (N = 21)	HBsAg negative (N = 3410)	P-value^b^
**Sex**				0.465
Male	1412/3431 (41.2 %)	7/21 (33.3 %)	1405/3410 (41.2 %)	
Female	2019/3431 (58.8 %)	14/21 (66.7 %)	2005/3410 (58.8 %)	
**Median age (IQR) in years**	22 (19, 26)	29 (25, 30)	22 (19, 26)	**<0.001**
**Age groups (in years)**				**<0.001**
15 - 19	1019/3431 (29.7 %)	0/21 (0.0 %)	1019/3410 (29.9 %)	
20 - 24	1295/3431 (37.8 %)	3/21 (14.3 %)	1292/3410 (37.9 %)	
25 - 30	1114/3431 (32.5 %)	18/21 (85.7 %)	1096/3410 (32.2 %)	
**Area of residence**				0.053
Rural	2712/3431 (79.0 %)	13/21 (61.9 %)	2699/3410 (79.1 %)	
Urban/Peri-urban	719/3431 (21 %)	8/21 (38.1 %)	711/3410 (20.9 %)	

HBsAg: Hepatitis B Virus surface antigen.

a Total population accepting HBsAg screening when offered as part of a combined sexual health/HIV assessment.

b Chi-squared or exact p-value as appropriate.

**Table 2:** Summary of recommendations for HBV interventions to be delivered through Sexual and Reproductive Health (SRH) programmes.

Domain	Recommendation
Offering HBV vaccination	• Incorporate catch-up vaccination for AYA in SRH programmes, where recommended and cost-effective (particularly for those at high risk of HBV exposure).
Tackling barriers to PrEP uptake and retention	• Conduct qualitative research to better understand barriers to PrEP adherence and retention (e.g. stigma, side effects, and logistical/economic challenges). • Evaluate role of community-based refill systems, peer support, and education campaigns. • Consider mobile health interventions, such as SMS reminders for refills and follow-up appointments, and telemedicine to provide remote consultations and support.
Providing HBV screening	• Assess HBV screening in HIV PrEP programmes in different settings, to improve HBV diagnosis and treatment uptake, particularly in high-prevalence regions. • Undertake cost-effectiveness evaluation of HBV screening and treatment through SRH/PrEP programmes.
Monitoring HBV Treatment long-term	• Evaluate long-term outcomes of HBV treatment through PrEP, including the risk of HBV flares during treatment interruptions and the impact of long-acting PrEP formulations.
Improving advocacy and funding	• Develop advocacy for sustained funding and policy support to ensure the continuity of access to treatment through cost-effective programmes in an uncertain funding landscape.
Promoting integration with ‘Triple Elimination’ agenda	• Integrate HBV with HIV and syphilis elimination efforts (the ‘triple elimination agenda’), to include combined education, screening, and treatment programmes.

AYA – adolescents and young adults; HBV – hepatitis B virus; HIV – human immunodeficiency virus; PrEP – pre-exposure prophylaxis; SRH – sexual and reproductive health.

### 3.2 Characteristics of individuals living with HBV

Compared to the HBV-negative population, the 21 AYA testing HBsAg-positive were comparable in sex (14/21, 66.7 % female vs 2005/3410, 58.8 % p = 0.47). Those testing HBsAg-positive were typically older than their HBsAg-negative counterparts (median age 29 years vs. 21 years respectively; p < 0.001) Table 1. The proportion of common curable STIs in AYA living with HBV was no different from those who tested HBsAg-negative (7/21 (33.3 %) vs 668/2293 (29.1 %); p = 0.67) (Suppl Table 1).

### 3.3 PrEP uptake in people living with HBV

The 21 individuals diagnosed with HBV had not previously been aware of their HBV status, but one was already receiving ART for treatment of HIV infection. The other 20 were counselled regarding starting oral PrEP, among whom 16/20 (80 %) were considered eligible for PrEP as an intervention for HIV prevention (Box 1). PrEP was taken up as a combined intervention for HBV treatment and HIV prophylaxis in 15/16 (94 %) of those eligible, while one individual decided not to take it. Individuals testing HBsAg-positive were more likely to be eligible for PrEP compared to their HBsAg-negative counterparts based on locally applied PrEP criteria (p = 0.02, Suppl Table 2). The uptake of PrEP was also significantly higher in people living with HBV infection compared to their HBV-negative counterparts (Suppl Table 2).

### 3.4 Continuity of PrEP access

When investigating follow up and retention in care, among the 15/15 (100 %) who were due for a refill, 8/15 (53.3 %) returned for at least one refill. Only 7/15 (46,7 %) returned for at least two refills over a median follow up time of 435 days (Fig. 2; Suppl Table 2).

### 3.5 Data access

Metadata records of our survey can be accessed through the AHRI repository using the following link: https://doi.org/10.23664/ahri.evolve.prep.

## 4 Discussion

This pilot study provides early evidence for the feasibility of providing an assessment of HBV screening and offering PrEP as part of a programme of SRH and HIV care for AYA in KZN, SA. WHO guidelines have been expanded with the goal of reaching 40 million more people with HBV treatment by the year 2026, and provide flexibility for use of dual therapy TDF/XTC.^2^ The use of TDF/XTC as a combination therapy for HIV PrEP and HBV treatment meets mandates for decentralisation and task-sharing,^3^ making interventions more accessible to wider populations, and with potential cost-savings for health systems. Capitalising on existing frameworks for HIV and SRH could improve pathways for diagnosis and access to treatment. Recommendations based on learning from this study are presented in Table 2.

We found a lower prevalence of active HBV infection (0.6 %) compared to HBV prevalence rates previously observed in individuals receiving HIV care, and high risk individuals (sex workers (SWs), men who have sex with men (MSM) and people who inject drugs (PWID)) in whom HBV prevalence is 8.5 % and 4 % respectively.^17^^,^^18^ Lower rates in the general population are in keeping with the success of HBV vaccine roll-out as part of the WHO Expanded Programme on Immunisation since the mid-1990's. An additional factor associated with low HBsAg seroprevalence may be related to the young age of the population in this study and thus more likely to have received HBV vaccination. However, the results also reflect gaps in population HBV immunity, with some AYA living with HBV, reflecting the lack of sufficient roll out of the birth dose vaccine, and possible gaps in coverage of the routine multivalent infant vaccine schedule. This also leaves a susceptible population, with the chance that new infections will be acquired over time, which (if progressing to chronicity) would increase the prevalence in older adults. Among the small number of diagnosed people living with HBV (PLWHB), there was a high uptake of antivirals, but a fall-off in PrEP continuity over time, as has been found in other studies.^19^^,^^20^

One individual who had tested HBsAg-positive and was deemed PrEP eligible declined this intervention. The motives for not wishing to take PrEP in this individual were not documented, but potential barriers to daily oral PrEP uptake in this study have included pill burden, fear of inadvertently disclosing sexual activity, stigma and concern about the side effects. Other barriers in the literature are doubting the efficacy of PrEP, size of the pill, myths and misconceptions about PrEP use, and costs of long-term access (e.g. travelling or missed time from work to collect refills).^21^

Sustainable access to PrEP has been thrown into question by the suspension of programmes delivered with federal funding from the United States in January 2025, with the long-term status of PEPFAR becoming insecure. In addition to the serious implications for increasing risk of HIV transmission, loss of access to PrEP may also influence access to HBV treatment.^22^

### 4.1 Caveats and limitations

The *Theta Nami* and *LAPIS* studies are successfully engaging a sexually active group with high PrEP eligibility. However, to date, we have only identified a small number of individuals who started PrEP as a combined intervention for HBV treatment and HIV prophylaxis. As numbers are small, we cannot comment robustly on rates of acceptance or long-term continuity of PrEP. Although all young people in the communities targeted can access the SRH clinics, our data are not fully representative of the whole community, as those who do not engage or opt out may have specific vulnerabilities potentially associated with increased risk of blood-borne virus infections.

While AYA born after 1995 should have received three doses of HBV vaccine through routine infant immunisation programmes, the coverage has been incomplete and population immunity is not secure. SRH programmes offer an opportunity to offer catch-up vaccination to those with risk-factors for HBV exposure. However, although HBV vaccines were offered to some individuals within the programme, to date this intervention has not been formally assessed and we are unable to report on the numbers who were eligible, offered, accepted or completed a vaccine schedule.

We used the Abbott assay for HBsAg detection, which provides a categorical assessment of HBsAg status. In this study we are unable to report quantitative results of HBV DNA viral load testing, as these data are not routinely available and were not costed as part of this study. However, scale-up of access to HBV DNA quantification is an important aspiration for routine clinical practice and for research studies, as viral load is part of the recommended risk-assessment for offering and monitoring antiviral treatment or prophylaxis.

We have not considered the cost-effectiveness of continued HBsAg screening in this young adult population; such evaluation would be needed to determine the appetite for public health programmes to continue HBV screening depending on local prevalence. However, including HBV testing in a population programme for SRH, in which the staff and infrastructure costs are already met, may be cost-effective even when the local population prevalence is low, as the additional cost of HBsAg screening is small, but leads to significant individual and public health benefits over the long-term.

### 4.2 Longer term aims

Future aims for HBV will include further investigating the community's acceptance of screening, treatment and preventive interventions for HBV with qualitative research to determine knowledge, beliefs and experiences, and to identify and tackle barriers. Factors that contribute to attrition from follow-up need to be studied further in order to improve continuity of PrEP, which is particularly important in those in whom this medication is being used for HBV treatment.

There is a need for continuous evaluation of the impact of shifting HIV interventions on the HBV treatment landscape, as HIV treatment moves towards dual therapy regimens (based on dolutegravir) and long-acting injectable PrEP. While dolutegravir in HIV regimens may be combined with the HBV-active agent lamivudine, the latter is associated with resistance in HBV and is therefore not a recommended option (unless in combination with another HBV-active agent such as tenofovir).^23^ Maintaining access to gold-standard HBV treatment is essential as HIV regimens change. Long-term adherence to PrEP for PLWHB is important, as there are concerns about the potential for flares of HBV associated with treatment interruptions.^24^ This is a potential risk for PrEP that contains HBV active agents (such as TDF/XTC), not for PrEP that does not have activity against HBV (such as injectable cabotegravir or lenacapavir); however, in practice serious adverse events have not been described on PrEP cessation in PLWHB.^4^

PrEP is primarily used for HIV prevention but could also be expanded to individuals at risk of HBV. The integration of SRH, HIV and HBV services offers opportunities to combine education, screening, family planning, delivery of perinatal care and prevention of mother to child transmission (PMTCT) in keeping with the global ‘triple elimination agenda’ for HIV, HBV and syphilis. This model offers an efficient use of resources that allows multiple overlapping health challenges to be tackled together. However, as the funding landscape changes, and PEPFAR provision was paused in January 2025, many such programmes are becoming highly vulnerable.^25^

## 5 Conclusion

The framework we present here highlights opportunities to shape and integrate HBV education, prevention, diagnosis and treatment into wider SRH/HIV services.^3^ PrEP may be a valuable way for delivering effective and consistent antiviral therapy to PLWHB, especially in settings in which infrastructure for delivery of viral hepatitis care is currently limited or absent.

## CRediT authorship contribution statement

**Gloria Sukali:** Writing – review & editing, Writing – original draft, Visualization, Validation, Project administration, Methodology, Investigation, Formal analysis, Data curation, Conceptualization. **Jacob Busang:** Writing – review & editing, Visualization, Validation, Methodology, Formal analysis, Data curation. **Jaco Dreyer:** Writing – review & editing, Project administration, Data curation. **Thandeka Khoza:** Writing – review & editing, Project administration. **Marion Delphin:** Writing – review & editing, Visualization, Validation, Methodology. **Nonhlanhla Okesola:** Writing – review & editing, Project administration. **Carina Herbst:** Writing – review & editing, Project administration. **Elizabeth Waddilove:** Writing – review & editing, Methodology. **Janine Upton:** Writing – review & editing. **Janet Seeley:** Writing – review & editing, Visualization, Validation, Methodology. **Collins Iwuji:** Writing – review & editing, Visualization, Validation, Supervision, Methodology. **Motswedi Anderson:** Writing – review & editing, Visualization, Validation, Project administration, Methodology. **Philippa C. Matthews:** Writing – review & editing, Visualization, Validation, Supervision, Resources, Project administration, Methodology, Funding acquisition, Data curation, Conceptualization. **Maryam Shahmanesh:** Writing – review & editing, Validation, Supervision, Resources, Project administration, Methodology, Investigation, Funding acquisition, Data curation, Conceptualization.

## Funding

PCM receives core funding from the 10.13039/100010438Francis Crick Institute (ref CC2223) which supports the EVOLVE-HBV project. PCM also receives funding support from the UCL/10.13039/501100008721UCLH 10.13039/100014461NIHR Biomedical Research Centre (BRC). GS is funded by a UCL-AHRI PhD fellowship. MS receives funding from 10.13039/100000865Bill and Melinda Gates Foundation INV-033650, INV-076790 and INV-076706; 10.13039/100000002US National Institute of Health (NIH) R01 (5R01MH114560-03); Africa Health Research Institute is the trial sponsor and is supported by core funding from the 10.13039/100010269Wellcome Trust strategic core award (201433/Z/16/A). MS is an NIHR Research Professor (NIHR 301634).

## Declaration of competing interest

PCM has received funding support from GSK for a doctoral fellow in her group and related to the UK NIHR Health Informatics Collaborative (outside the scope of the current work). The other authors declare that they have no known competing financial interests or personal relationships that could have appeared to influence the work reported in this paper.

## Data Availability

Data are archived in AHRI repository: https://doi.org/10.23664/ahri.evolve.prep
