# Supplementary material for: HIV PrEP programmes as a framework for diagnosing and treating HBV infection in adolescents and young adults in KwaZulu-Natal, South Africa
Source: J Virus Erad. 2025 Jun 6;11(3):100600. doi: 10.1016/j.jve.2025.100600 (PMC12213953; doi:10.1016/j.jve.2025.100600)
Supplement: Multimedia component 1 [file mmc1.pdf]

SUPPLEMENTARY MATERIAL

HIV PrEP programmes as a framework for diagnosing and treating HBV infection in adolescents and young adults in KwaZulu-Natal, South Africa

Gloria Sukali et al.

Supplementary methods

Storage and collection of samples for sexually transmitted infection:

Specimen collection kits were kept at ambient range (18 - 25°C). These kits were transported in a cooler box with ice bricks when nurses were going to different collection sites. POCT (for syphilis and HBsAg) was done from a finger-prick by the nurse at the site of clinical review and recruitment.

Vaginal swab and urine samples were transported in a cooler box with ice bricks to the Somkhele laboratory. Urine was aliquoted into Xpert transport collection tubes and then stored refrigerated (2 to 8°C). Vaginal swab samples were collected into Xpert transport tubes (at the clinic) and then refrigerated overnight in the Somkhele laboratory. Both urine and swabs were shipped to Durban refrigerated; once received in Durban, these samples were frozen (-20°C) until testing.

Supplementary Tables:

Supplementary Table 1: Relationship between hepatitis B surface antigen (HBsAg) status and sexually transmitted infections (STIs) in a cohort of adults and young adults attending clinical review for sexual and reproductive health and HIV risk assessment.

STI Characteristics	Total N=3,431	HBsAg positive N=21	HBsAg negative N=3,410	P-value†
**Any STI***				0.673
No	1639/2314 (70.8%)	14/21 (66.7%)	1625/2293 (70.9%)	
Yes	675/2314 (29.2%)	7/21 (33.3%)	668/2293 (29.1%)	
**Chlamydia**				0.302
No	1762/2312 (76.2%)	14/21 (66.7%)	1748/2291 (76.3%)	
Yes	550/2312 (23.8%)	7/21 (33.3%)	543/2291 (23.7%)	
**Gonorrhoea**				0.162
No	2157/2312 (93.3%)	18/21 (85.7%)	2139/2291 (93.4%)	
Yes	155/2312 (6.7%)	3/21 (14.3%)	152/2291 (6.6%)	
**Trichomonas‡**				0.618
No	1408/1513 (93.1%)	15/15 (100.0%)	1393/1498 (93.0%)	
Yes	105/1513 (6.9%)	0/15 (0.0%)	105/1498 (7.0%)	
**Syphilis**				0.293
Negative	3374/3430 (98.4%)	20/21 (95.2%)	3354/3409 (98.4%)	
Positive	56/3430 (1.6%)	1/21 (4.8%)	55/3409 (1.6%)	
**Ever treated any STI**				0.609
No	115/675 (17.0%)	0/7 (0.0%)	115/668 (17.2%)	
Yes	560/675 (83.0%)	7/7 (100.0%)	553/668 (82.8%)	

† Chi-squared or exact p-value

* Any STI is the combination of common curable STIs in our setting (Chlamydia, Gonorrhoea, Trichomonas and Syphilis

‡ Trichomonas is now being tested among female participants only

Supplementary Table 2: PrEP uptake and retention according to Hepatitis B surface antigen (HBsAg) status among adolescent and young adult participants of sexual health screening and prevention programmes in KwaZulu Natal, South Africa

	Total individuals N=3,431	Individuals testing HBsAg-positive N=21	Individuals testing HBsAg-negative N=3,410	P-value*
**Assessed for PrEP eligibility**				1.000
No	289 (8.4%)	1 (4.8%)	288 (8.4%)	
Yes	3142 (91.6%)	20 (95.2%)	3122 (91.6%)	
**Ever eligible for PrEP**				0.022
No	1467/3142 (46.7%)	4/20 (20.0%)	1463/3122 (46.9%)	
Yes	1675/3142 (53.3%)	16/20 (80.0%)	1659/3122 (53.1%)	
**Ever started PrEP**				0.008
No	638/1675 (38.1%)	1/16 (6.2%)	637/1659 (38.4%)	
Yes	1037/1675 (61.9%)	15/16 (93.8%)	1022/1659 (61.6%)	
**Due for first PrEP refill**				0.100
No	1/1037 (0.1%)	0/15 (0%)	1/1022 (0.1%)	
Yes	1036/1037 (99.9%)	15/15 (100%)	1021/1022 (99.9%)	
**At least one PrEP refill**				0.258
No	630/1036 (60.8%)	7/15 (46.7%)	623/1021 (61.0%)	
Yes	406/1036 (39.2%)	8/15 (53.3%)	398/1021 (39.0%)	
**At least two PrEP refills†**				0.003
No	855/1036 (82.5%)	8/15 (53.3%)	847/1021 (83.0%)	
Yes	181/1036 (17.5%)	7/15 (46.7%)	174/1021 (17.0%)	
**Observation period**				
**Attended clinic at least twice**				0.029
No	2247/3431 (65.5%)	9/21 (42.9%)	2238/3410 (65.6%)	
Yes	1184/3431 (34.5%)	12/21 (57.1%)	1172/3410 (34.4%)	
**Clinical cohort duration (days)‡**				
Mean (min, max)	246 (1, 786)	418 (28, 672)	244 (1, 786)	0.002
Median (IQR)	195 (84, 365)	435 (325, 589)	194 (84, 364)	0.005
**PrEP cohort duration (days)**				
Mean (min, max)	79 (0, 920)	242 (0, 648)	77 (0, 920)	<0.001
Median (IQR)	15 (0, 84)	308 (0, 420)	14 (0, 84)	0.007
**Retention on PrEP (days) ****				
Mean (min, max)	178 (13, 920)	362 (84, 648)	175 (13, 920)	0.003
Median (IQR)	111 (49, 254)	371 (235, 477)	111 (46, 252)	0.004

HBsAg: Hepatitis B Virus surface antigen; PrEP: Pre-exposure Prophylaxis.

* Chi-squared or exact test p-value for categorical variables. T-test or ranksum test p-value for continuous variables

† The denominator reflects those who were due for their second PrEP refills.

‡ Clinical cohort duration includes participants who attended clinic at least twice (N=1184). It is calculated from the date of the first clinic visit to the date of the last clinic visit.

§ PrEP cohort duration includes participants who were ever on PrEP and due for a refill (N=1036). It is calculated from the date of the initial PrEP prescription to the date of the last PrEP prescription or self-reported stop date.

** Retention on PrEP is restricted to N=406 with at least two PrEP refills. It is calculated from the date of initial PrEP prescription to the date of the last PrEP prescription or self-reported stop date.
